# Functional poly(ionic liquid) with unique zwitterionic structure as efficient catalyst for the conversion of ethylene carbonate to dimethyl carbonate

**DOI:** 10.1002/smo.20240046

**Published:** 2025-01-02

**Authors:** Zhaoyang Qi, Fuying Zhang, Huiyun Su, Changshen Ye, Qinglian Wang, Ting Qiu, Jie Chen

**Affiliations:** ^1^ College of Chemical Engineering Fuzhou University Fuzhou Fujian China; ^2^ Qingyuan Innovation Laboratory Quanzhou China; ^3^ Fuzhou University International Joint Laboratory of Thermochemical Conversion of Biomass Fuzhou Fujian China

**Keywords:** anion and cation regulation, dimethyl carbonate production, mechanism, poly(ionic liquid)s, transesterification reaction

## Abstract

Dimethyl carbonate (DMC) is an important chemical raw material extensively used in organic synthesis, lithium‐ion battery electrolytes, *etc*. The primary method for industrial synthesis of DMC involves transesterification between ethylene carbonate and MeOH but faces issues with difficult catalyst separation and low catalytic activity. Based on the synergistic catalytic activity of cation and anion, this study develops poly(ionic liquid)s of [N_X_PIL][PHO] and [N_3_PIL][Y] with varying alkaline sites and alkalinity levels. This is accomplished by constructing functional polymer monomers containing free radical polymerization sites and nitrogen‐containing alkaline groups, and by polymerizing them with suitable cross‐linking monomers in a specific ratio before exchanging the resulting polymers with different anions. Results show that doping with nitrogen‐containing alkaline groups leads to enhanced basic functional sites while appropriate anions provide intensified alkalinity levels. The [N_3_PIL][PHO] obtained exhibits superior catalytic activity in transesterification synthesis of DMC, with a yield of 91.43% and selectivity of 99.96% at a reaction time of 2 h. The study also investigates the impact of poly(ionic liquid) cationic structure and anion types, as well as their interactions, on catalytic performance. The findings reveal that the catalytic activity of poly(ionic liquid) is restricted by the interactions between cation and anion. Based on these findings, a possible reaction mechanism was proposed, providing theoretical support for the high‐efficiency production of DMC.

## Graphical abstract

Based on the principle of synergetic catalysis by anions and cations in poly(ionic liquid), the catalysts suitable for transesterification synthesis of DMC by adjusting the number of nitrogen‐containing basic group monomers and anions with different basicities were prepared. The impact mechanism of doping cationic scaffolds with basic groups and anion types, as well as their interactions on catalytic performance was analyzed.

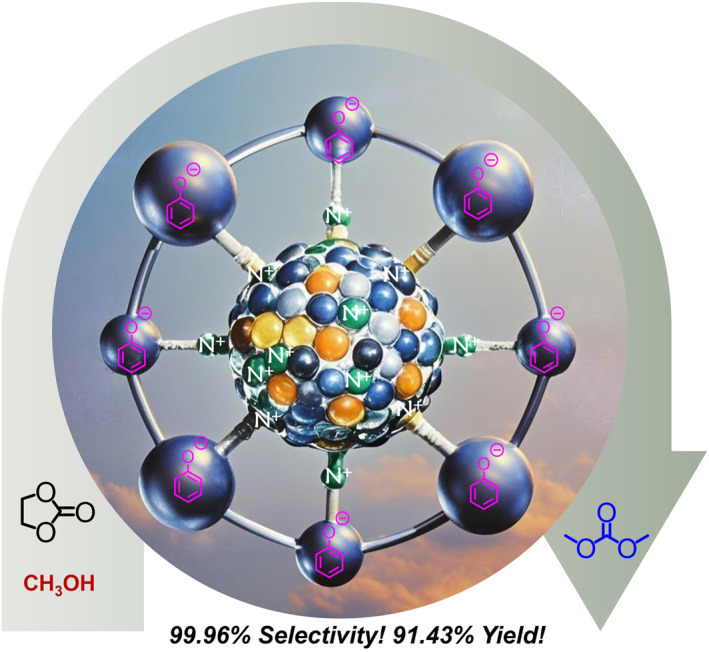

## 1 INTRODUCTION

Amidst the sustained development of the global economy, humanity faces the dual challenges of an energy crisis and environmental pollution. The traditional chemical industry is confronted with unprecedented challenges, leading to a widespread embrace of the concepts of green, economical, and sustainable chemical engineering. There is a growing emphasis on minimizing environmental pollution since the inception of industrial synthesis. Therefore, it is of paramount importance to seek low‐pollution chemical intermediates as substitutes for traditional high‐risk chemicals. Dimethyl carbonate (DMC), as an environmentally friendly chemical intermediate, is characterized by its green, non‐toxic, and easily biodegradable properties.^[1]^ These features have led to its widespread application in high‐performance resins, solvents, fuel additives, lithium‐ion battery electrolytes, and various other fields.^[2]^

At present, there are five industrial routes for synthesizing DMC: phosgene method, carbonylation of MeOH, urea alcoholysis, direct synthesis of MeOH and CO_2_, and transesterification method. However, the raw materials and intermediate products used in the phosgene method are toxic and carcinogenic.^[3]^ Additionally, the byproduct HCl produced from the phosgene method and the carbonylation of MeOH method can cause strong corrosion to the production equipment.^[4]^ Direct synthesis of DMC from CO_2_ and MeOH is hindered by thermodynamic constraints, resulting in low DMC yield.^[5]^ Urea alcoholysis faces difficulties in the forward reaction, and the yield of DMC is severely limited by the chemical equilibrium. In contrast, the transesterification is the most promising out of all the synthetic routes due to its low corrosiveness to equipment, easy process, and absence of waste emissions.[^6^, ^7^] This method aligns well with the sustainable development concept of “green and economical” production, and has become the current hot spot of DMC production process research.^[8]^

In industrial processes, DMC is mainly synthesized from ethylene carbonate (EC) and MeOH using methoxide as a homogeneous catalyst. However, the use of methoxide presents challenges in terms of difficult recovery post‐reaction, leading to increased production costs and substantial solid waste, thus limiting its application.^[9]^ With the introduction of sustainable development strategies, environmentally friendly alkaline heterogeneous catalysts have been widely researched due to their high catalytic activity, mild reaction conditions, ease of product separation post‐reaction, and low process costs. Nevertheless, currently reported alkaline heterogeneous catalysts still face issues such as easy loss of active components, poor thermal stability, and low catalytic activity.[^10^, ^11^, ^12^] Hence, the focal point of current research for DMC synthesis via the transesterification reaction lies in developing alkaline heterogeneous catalysts characterized by excellent catalytic stability and high activity.

Poly(ionic liquid)s are a class of polymers that feature ionic liquids as their repeating units.[^13^, ^14^, ^15^] Due to their tunable structure, chemical and thermal stability, and the mechanical robustness of polymers, they find extensive applications in the field of catalysis.^[16]^ Compared to homogeneous catalysts, heterogeneous poly(ionic liquid)s catalysts exhibit lower catalytic activity as the active sites are less exposed to the reactants.^[17]^ To address this issue, our research group designed a series of poly(ionic liquid)s that can swell in the presence of reactants. The swelling of these poly(ionic liquid)s allows a substantial amount of reactant to penetrate into the polymer matrix, promoting intimate contact between reactants and the active sites, thereby achieving a quasi‐homogeneous catalytic effect.^[18]^ In particular, for the transesterification between EC and MeOH to synthesize DMC, we synthesized various basic poly(ionic liquid)s catalysts with excellent swelling properties in the reaction system, which exhibited notable catalytic performance. However, there remains room to further enhance their activity. Analysis of the EC and MeOH transesterification process reveals it to be a typical nucleophilic addition‐elimination reaction.[^19^, ^20^] The key to this reaction lies in the formation of a methoxide ion (CH_3_O^−^) through the abstraction of an electron by a basic site. This CH_3_O^−^ then attacks the positively charged carbonyl carbon of EC, resulting in the formation of DMC. Furthermore, the efficiency of the basic ionic liquid catalyst in this reaction is attributed to the synergistic catalytic action of the anions and cations: the protonated cation activates EC through interaction with the carbonyl group, while the anion provides sufficient basicity to activate MeOH, generating CH_3_O^−^. This synergy between ions effectively catalyzes the transesterification process. Studies have also shown that the catalytic activity of transesterification materials is closely related to their basicity. Therefore, investigating the structure and properties of the ionic components to understand their impact on the basicity of poly(ionic liquid)s will aid in the development of highly active and selective catalytic materials for transesterification.

Based on the synergistic catalytic effect of cation and anion in transesterification reaction in ionic liquids, a series of functionalized poly(ionic liquid)s were designed and prepared by adjusting the structure of cationic and anionic moieties through the tunable structural characteristics of poly(ionic liquid)s. The alkaline sites and amounts of poly(ionic liquid)s were modulated accordingly. These functionalized poly(ionic liquid)s, with varying numbers of nitrogen‐containing alkaline groups, were evaluated and used for the catalysis of the transesterification between EC and MeOH. The effects of nitrogen‐doped alkaline groups on the catalytic performance of poly(ionic liquid)s were investigated systematically. Additionally, the effects of different anionic functional groups on the catalytic performance of poly(ionic liquid)s were explored. The reaction conditions were optimized to achieve the best reaction results. Finally, an in‐depth study on the reaction mechanism of poly(ionic liquid)‐catalyzed synthesis of DMC was conducted, providing theoretical support for the efficient preparation of DMC.

## 2 EXPERIMENTAL

The materials utilized in this study are delineated in the Supporting Information, and all chemicals were employed without subsequent purification.

### 2.1 Catalyst preparation

**FIGURE 1:**
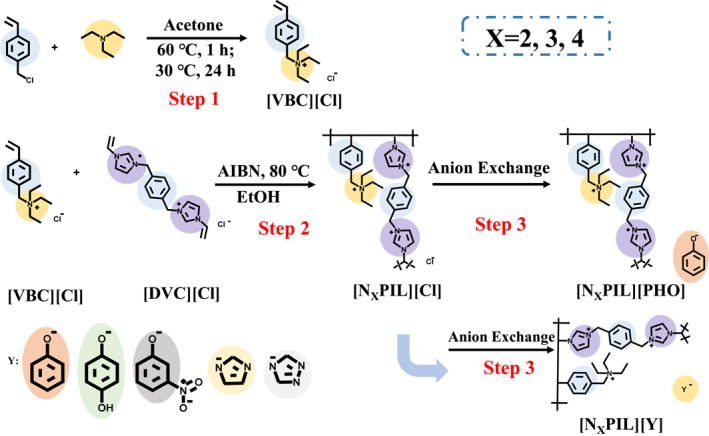
The synthesis route of [N_X_PIL][Y] with different N content and anions.

An array of alkaline poly(ionic liquid) was constructed by introducing nitrogen‐containing basic groups into the cationic framework and exchanging different anions, thereby establishing a controlled number of basic sites within the poly(ionic liquid). The specific synthesis process, as illustrated in Figure 1, involved the utilization of triethylamine as the nitrogen‐containing base, which was selectively grafted onto 4‐vinylbenzyl chloride containing vinyl groups, thereby preparing monomers with polymerization sites and nitrogen‐containing basic groups. Subsequently, these monomers were added in a predetermined proportion to the previously synthesized cross‐linked framework monomer 1, 4‐di(chloro‐1‐methylene‐3‐vinylimidazole)benzene, designated as [DVC][Cl] and underwent free‐radical polymerization under the influence of an initiator, thus producing a series of poly(ionic liquid) with varying nitrogen‐containing basic group content. Following, an ion exchange process was employed to exchange the anions within the aforementioned poly(ionic liquid) with phenol (PHO), imidazole (IM), 1, 2, 4‐triazole (TRIZ), 2‐hydroxypyridine (OP), nitrophenol (NPHO), and hydroquinone (2PHO), thereby yielding alkaline poly(ionic liquid) catalytic materials with different anionic functional groups.

#### 2.1.1 Synthesis of [VBC][Cl]

5 ml of triethylamine was dissolved in acetone and placed in a Schlenk reaction tube. Then, 5 ml of 4‐vinylbenzyl chloride was slowly added (0.5 ml/min) to the reaction tube dropwise. The mixture was heated to 60°C and stirred (200 r/min) for 1 h. Afterward, the temperature was lowered to 30°C and the reaction was continued for 24 h. At the end of the reaction, the resulting white solid was washed with 50 ml of ice‐cold isopropanol three times to remove any residual triethylamine and 4‐vinylbenzyl chloride. Finally, the solid product with polymerization sites and nitrogen‐containing basic groups, 1, 4‐(1‐methyl‐vinyl)‐chloride‐4‐triethylammonium benzene, was obtained and denoted as [VBC][Cl].

#### 2.1.2 Synthesis of [N_X_PIL][Cl]

1.0 mmol of 1,4‐dichlorobenzyl bearing two anchoring sites, along with 2.0 mmol of 1‐vinylimidazole, was dissolved in 100 ml of acetonitrile. After dissolution, the mixture was placed in a Schlenk reaction flask and heated to 60°C and left to react for 24 h. Upon completion of the reaction, the resulting white solid powder was washed with 50 ml of acetonitrile three times to remove any residual 1,4‐dichlorobenzyl and 1‐vinylimidazole. Subsequently, vacuum freeze‐drying was performed to obtain a polymeric monomer 1,4‐bis(chloromethyl‐1‐ethylimidazolium)benzene functionalized with the imidazolium moiety, denoted as [DVC][Cl] (^1^H NMR (600 MHz, MeOD) δ 8.08 (d, J = 2.2 Hz, 2H, CH), 7.79 (d, J = 2.2 Hz, 2H, CH), 7.61 (s, 4H, Ar‐H), 7.31 (dd, J = 15.6, 8.7 Hz, 2H, CH), 5.97 (dd, J = 15.6, 2.8 Hz, 2H, CH), 5.56 (s, 4H, CH_2_), 5.48 (dd, J = 8.7, 2.8 Hz, 2H, CH)). The [DVC][Cl] and [VBC][Cl] were dissolved in ethanol (10 times the mass of the [DVC][Cl]) in ratios of 2:1, 3:1, and 4:1. The resulting solutions were then placed in a high‐pressure vacuum reaction vessel, and AIBN was added. The reaction vessel was sealed with N_2_ and heated to 80°C for 24 h. Upon completion of the reaction, the resulting yellowish gel‐like solid was washed with 50 ml of deionized water for three times to remove any remaining [DVC][Cl] and [VBC][Cl]. Subsequently, the solid was vacuum‐dried to yield copolymers of [DVC][Cl] and [VBC][Cl], denoted as [N_X_PIL][Cl] (*X* = 2, 3, 4).

#### 2.1.3 Synthesis of [N_3_PIL][Y]

The [N_3_PIL][Cl] (20 g) was introduced into a column and a 1 mol/L sodium hydroxide solution (200 ml) was added to allow slow dripping. The eluent was monitored with a standard silver nitrate solution until it became transparent. The resulting polymer was then dispersed in 200 ml of deionized water. Subsequently, PHO, TRIZ, IM, NPHO, and 2PHO (all are 20 g) were separately added to ensure complete dissolution in the water. After shaking in a shaker for 24 h, the mixture was extracted, washed with 50 ml of hot deionized water three times to eliminate residual acid, and then subjected to vacuum drying, resulting in the alkaline poly(ionic liquid) denoted as [N_3_PIL][Y].

The methods for structural analysis of the aforementioned synthesized various poly(ionic liquid)s and precursors can be found in the Supporting Information.

### 2.2 Catalytic reactions

The catalytic activity was evaluated using the batch experiments, and DMC yield and selectivity were determined under the following conditions: reaction temperature of 70°C, reaction time of 10 h, catalyst dosage of 2 wt.% (based on EC), and molar ratio of n(MeOH):n(EC) = 10. Subsequently, upon completion of the reaction, the mixture was cooled using ice water, and the product was analyzed by gas chromatography (GC2014 Shimadzu, Flame Ionization Detector, HP‐5 capillary column, 30 m × 0.32 mm × 0.25 μm), with n‐BuOH utilized as an internal standard. The EC conversion (%), DMC selectivity (%), and DMC yield (%) were calculated using the following formulae:

(1) $$X_{\text{EC}}=\frac{n_{0\text{EC}}-n_{\text{EC}}}{n_{0\text{EC}}}\times 100\%$$

(2) $$Y_{\text{DMC}}=\frac{n_{\text{DMC}}}{n_{0\text{EC}}}\times 100\%$$

(3) $$S_{\text{DMC}}=\frac{Y_{\text{DMC}}}{X_{\text{EC}}}\times 100\%$$

Within the equation, *n*_*0*__*EC*_ denotes the initial molar quantity of EC, while *n*_*EC*_ denotes the molar quantity after the reaction at time *t*; *n*_DMC_ signifies the molar quantity of the resulting product, DMC. Evaluating its recyclability, the solid catalyst after the initial reaction was separated from the reaction medium and subsequently employed for the successive transesterification reaction cycle.

## 3 RESULTS AND DISCUSSION

### 3.1 Characterization

The nuclear magnetic resonance spectroscopy (NMR) spectrum of the [VBC][Cl] monomer is depicted in Figure S1. The peaks corresponding to the benzene ring of the 4‐vinylbenzyl chloride are discernible, specifically a doublet at 7.51 ppm and another doublet at 7.41 ppm. Additionally, peaks representing triethylamine are observed depicted as a triplet at 3.14 ppm and another triplet at 1.30 ppm. Importantly, the integrated curves of these peaks, once normalized, exhibit a 1:1, indicating the successful attachment of triethylamine to the 4‐vinylbenzyl chloride. The nuclear magnetic resonance data is as follows: ^1^H NMR (600 MHz, D_2_O) δ 7.51 (d, J = 8.2 Hz, 2H, Ar‐H), 7.41 (d, J = 8.2 Hz, 2H, Ar‐H), 6.74 (dd, J = 17.7, 11.0 Hz, 1H, CH), 5.85 (d, J = 17.7 Hz, 1H, CH), 5.32 (d, J = 11.0 Hz, 1H, CH), 4.31 (s, 2H, CH_2_), 3.14 (q, J = 7.2 Hz, 6H, 3CH_2_), 1.30 (t, J = 7.2 Hz, 9H, 3CH_3_).

**FIGURE 2:**
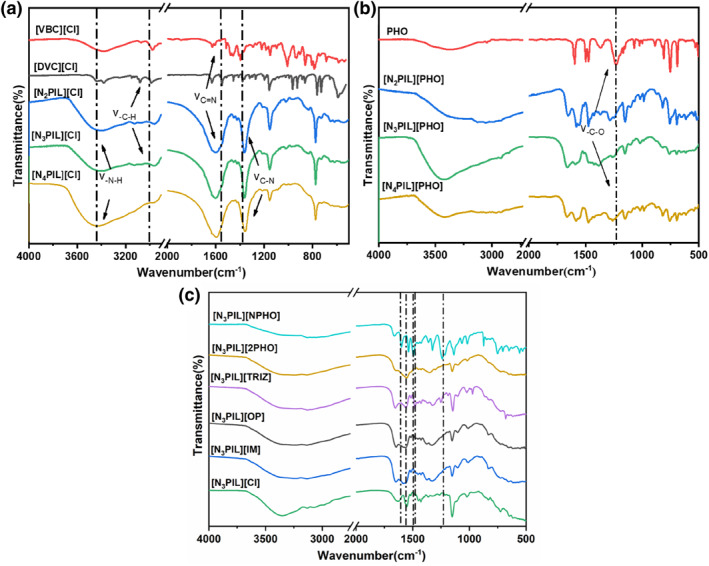
FT‐IR spectra of the basic poly(ionic liquid)s [N_X_PIL][Cl], [N_X_PIL][PHO] and [N_3_PIL][Y]. FT‐IR, fourier transform infrared spectra.

To ascertain the successful synthesis of the poly(ionic liquid)s, fourier transform infrared spectra (FT‐IR) studies were initially conducted on the monomer [VBC][Cl], as well as the poly(ionic liquid)s [N_X_PIL][Cl], [N_X_PIL][PHO], and [N_3_PIL][Y] before and after anion exchange. The results are depicted in Figure 2. The FT‐IR spectrum of [VBC][Cl] exhibits characteristic vibrations at 686 cm^−1^, corresponding to the out‐of‐plane bending vibration of the aromatic C‐H bond, and at 1007 cm^−1^, signifying the stretching vibration of the quaternary amine present in [VBC][Cl], confirming the successful synthesis of [VBC][Cl]. In the spectra of [N_2_PIL][Cl], [N_3_PIL][Cl], and [N_4_PIL][Cl], distinct vibrations are observed at 3444 cm^−1^, 1555 cm^−1^, and 1375 cm^−1^, corresponding to the N‐H bond and the C = N, C‐N vibrations of the imidazole ring.[^21^, ^22^] Additionally, a vibration at 1007 cm^−1^ corresponding to the quaternary ammonium group on [VBC][Cl] is observed, with the intensity of this vibration increasing gradually with the augmentation of the [VBC][Cl] addition, indicating the successful preparation of [N_2_PIL][Cl], [N_3_PIL][Cl], and [N_4_PIL][Cl] via free radical polymerization to introduce different proportions of nitrogen‐containing basic groups. Furthermore, post phenol anion exchange, the FT‐IR spectra of [N_2_PIL][PHO], [N_3_PIL][PHO], and [N_4_PIL][PHO] reveal a distinctive stretching vibration peak at 1226 cm^−1^, indicative of the ‐C‐O stretching vibration on the phenol moiety. This signifies the successful exchange of the phenol anion into the aforementioned poly(ionic liquid)s, confirming the successful synthesis of [N_2_PIL][PHO], [N_3_PIL][PHO], and [N_4_PIL][PHO].

The FT‐IR spectrum in Figure 2c represents the alkaline poly(ionic liquid) [N_3_PIL][Y] containing different anions. In comparison to [N_3_PIL][Cl], [N_3_PIL][NPHO] exhibits a stretching vibration peak at 1496 cm^−1^, indicative of the ‐N = O functionality, confirming the successful mounting of the [NPHO]^‐^ anion through anion exchange onto the alkaline poly(ionic liquid). In the FT‐IR spectrum of [N_3_PIL][IM], the vibration peak of the imidazole ring at 1610 cm^−1^ is noticeably intensified compared to [N_3_PIL][Cl], indicating the successful exchange of the [IM]^‐^ anion. For [N_3_PIL][OP] and [N_3_PIL][TRIZ], the FT‐IR spectra reveal characteristic vibrations at 1574 cm^−1^ representative of the pyridine ring, and at 1481 cm^−1^ corresponding to the N‐N stretching vibration, validating the successful exchange of the [OP]^‐^ and [TRIZ]^‐^ anions into the poly(ionic liquid). In these spectra, the characteristic peak representing the quaternary ammonium at around 1007 cm^−1^ shifts to approximately 1022 cm^−1^ after ion exchange. This shift suggests a certain interaction between the anionic and cationic species within the poly(ionic liquid), indicating a crucial condition for stabilizing catalytic processes.

**FIGURE 3:**
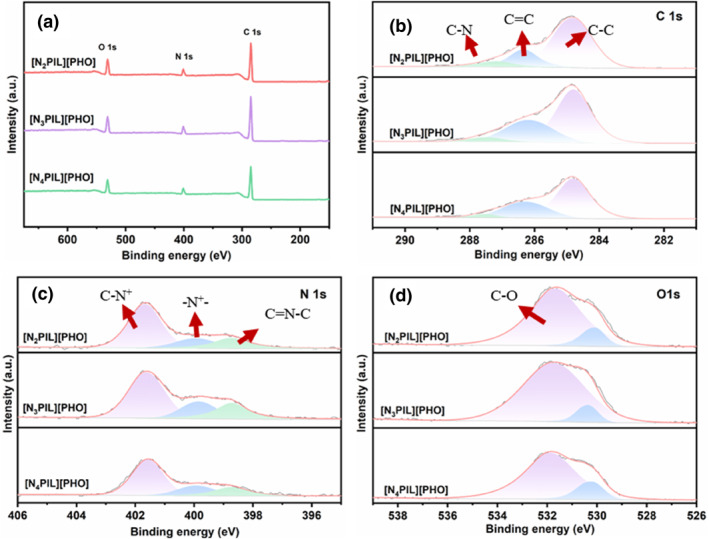
XPS spectra of basic poly(ionic liquid)s [N_2_PIL][PHO], [N_3_PIL][PHO], [N_4_PIL][PHO]: (a) Full‐scale spectra; (b) C 1s spectra; (c) N 1s spectra; (d) O 1s spectra. XPS, X‐ray photoelectron spectroscopy.

The X‐ray photoelectron spectroscopy (XPS) survey spectra of [N_2_PIL][PHO], [N_3_PIL][PHO], and [N_4_PIL][PHO] were analyzed, and shown in Figure 3. The [N_2_PIL][PHO], [N_3_PIL][PHO], and [N_4_PIL][PHO] all exhibit signals for the elements C, N, and O, without any signal for Cl, indicating the successful synthesis of the poly(ionic liquid) and the effective exchange of the phenolate anion. Further analysis of the XPS spectra of the three poly(ionic liquid)s reveals characteristic peaks in the C 1s spectrum at 284.8 eV, 286.2 eV, and 287.5 eV, corresponding to C‐C, C = C, and C‐N bonds, confirming the presence of the imidazole functional group.^[23]^ In the N 1s spectrum, the peaks at 398.7 eV, 399.8 eV, and 401.6 eV are attributed to the C = N‐C, ‐N^+^‐, and C‐N^+^ bonds, demonstrating the successful attachment of the imidazole ring and triethylamine.^[24]^ Additionally, the peak area contents of ‐N^+^‐ and C‐N^+^ bonds of [N_2_PIL][PHO] (0.17, 0.61) were similar to those of [N_3_PIL][PHO] (0.21, 0.55) and [N_4_PIL][PHO] (0.19, 0.63), indicating that the number of exchangeable active sites was similar (Table S1). The O 1s spectrum displays a peak at 531.7 eV attributed to the C‐O bond in the [PHO]^‐^ functional group, further confirming the presence of the phenolate anion. In conclusion, according to the XPS results, it is further evidenced that the imidazole ring and triethylamine were not compromised during the synthesis of the poly(ionic liquid) or the anion exchange process, and the phenolate anion was successfully exchanged into the poly(ionic liquid)s [N_2_PIL][PHO], [N_3_PIL][PHO], and [N_4_PIL][PHO].

The ^13^C NMR spectra of the poly(ionic liquid)s [N_2_PIL][PHO], [N_3_PIL][PHO], and [N_4_PIL][PHO] are presented in Figure S2. All three poly(ionic liquid)s exhibit signal peaks at 52.83 and 7.85 ppm, respectively, representing the ‐CH_2_‐ and ‐CH_3_ of triethylamine. Signal peaks at 117.45 and 130.17 ppm are attributed to the imidazole ring and the phenyl ring, respectively. Moreover, signal peaks at 161.38 ppm representing the ‐C‐O in the phenol anion were observed in the ^13^C NMR spectra of [N_2_PIL][PHO], [N_3_PIL][PHO], and [N_4_PIL][PHO], confirming the successful synthesis of the poly(ionic liquid)s [N_2_PIL][PHO], [N_3_PIL][PHO], and [N_4_PIL][PHO].

**FIGURE 4:**
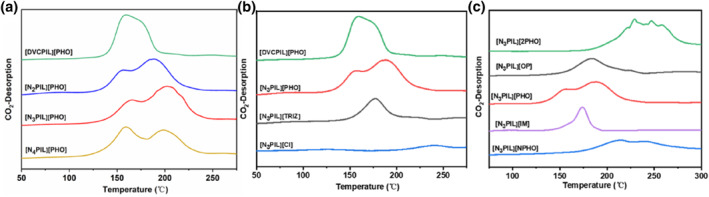
CO_2_‐TPD profiles of the basic poly(ionic liquid)s [N_X_PIL][Cl], [N_X_PIL][PHO] and [N_3_PIL][Y].

To delve further into the influence of doping nitrogen‐containing alkaline groups and exchanging different types of anions on the alkalinity of poly(ionic liquid)s, CO_2_‐TPD analysis was carried out with results presented in Figure 4. The TPD spectrum of [DVCPIL][PHO], which lacks nitrogen‐containing alkaline groups, reveals a single peak at 157°C, indicating the presence of only one alkaline site on its surface.[^25^, ^26^, ^27^, ^28^] Conversely, for the nitrogen‐containing alkaline groups doped [N_2_PIL][PHO], [N_3_PIL][PHO], and [N_4_PIL][PHO], a new desorption peak appears at 188°C in the CO_2_‐TPD spectra, signaling the existence of two distinct intensities of alkaline sites. Moreover, the introduction of nitrogen‐containing alkaline groups bestows the poly(ionic liquid)s with stronger alkaline sites. The desorption peaks observed around 188°C for [N_4_PIL][PHO] and [N_3_PIL][PHO] are slightly smaller than that of [N_2_PIL][PHO], as indicated by the integrated peak areas in Table S2, affirming the successful synthesis of poly(ionic liquid)s doped with varying proportions of nitrogen‐containing alkaline groups.

Furthermore, in investigating the alkalinity of poly(ionic liquid)s when exchanging different anions, as depicted in Figure 4, it is evident that the CO_2_‐TPD spectra of [N_3_PIL][Cl], which lacks basic anionic functional groups, does not exhibit characteristic absorption peaks, indicating the absence of alkaline sites in the non‐functionalized poly(ionic liquid)s. When poly(ionic liquid)s exchange different basic anionic functional groups, the integrated peak areas of the CO_2_‐TPD spectra, as shown in Table S3, follow the order: [N_3_PIL][2PHO] > [N_3_PIL][PHO] > [N_3_PIL][NPHO] > [N_3_PIL][OP] > [N_3_PIL][IM] > [N_3_PIL][TRIZ] > [N_3_PIL][Cl]. This sequence demonstrates that the alkalinity of poly(ionic liquid)s can be modulated through basic anionic functional groups, thus providing a basis for the precise regulation of the transesterification reactivity of poly(ionic liquid)s.

**FIGURE 5:**
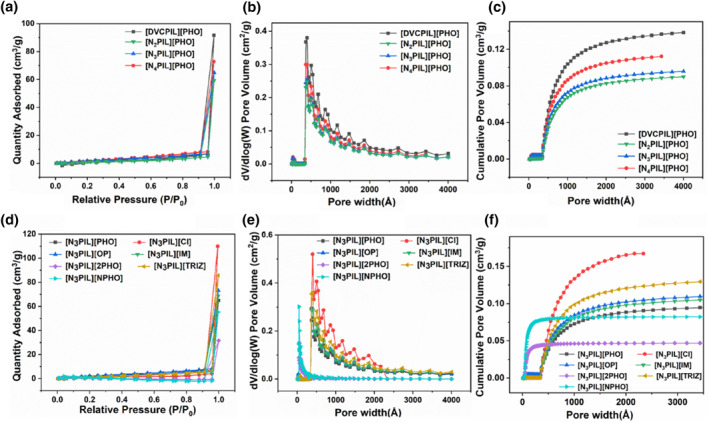
(a) N_2_ sorption isotherms, (b) pore size distribution and (c) pore volume distribution of basic poly(ionic liquid)s [N_X_PIL] [PHO]; (d) N_2_ sorption isotherms, (e) pore size distribution and (f) pore volume distribution of basic poly(ionic liquid)s [N_3_PIL][Y].

An analysis of the specific surface area and pore size distribution of the prepared alkaline poly(ionic liquid)s was conducted, with results presented in Figure 5 and Tables S4 and S5. As the doping number of nitrogen‐containing alkaline groups increases, the specific surface area reduces from 35 m^2^/g to 5 m^2^/g. This phenomenon stems from the fact that poly(ionic liquid)s are composed of cross‐linking monomers and polymerizable functional monomers, where the introduction of nitrogen‐containing alkaline groups reduces the content of cross‐linking monomers. This diminishes the cross‐linking density of the poly(ionic liquid)s, thereby affecting its molecular and pore structure and leading to a decrease in specific surface area. To investigate the impact of nitrogen‐containing alkaline groups on the pore structure of poly(ionic liquid)s, the cumulative pore volume of the synthesized poly(ionic liquid)s was measured. From Figure 5, it is observed that compared to the nitrogen‐free [DVCPIL][PHO], the pore volume of the poly(ionic liquid) gradually decreases from 0.052 cm^3^/g to 0.034 cm^3^/g upon the introduction of nitrogen‐containing alkaline groups. This reduction may be attributed to the partial blockage of the cationic framework's pores due to the doping of nitrogen‐containing alkaline groups. Furthermore, with increasing doping levels, the pore size of the poly(ionic liquid)s gradually decreases, albeit remaining larger than 3.5 nm, which is advantageous for mass transfer during the reaction process. Moreover, the specific surface area and pore volume of alkaline poly(ionic liquid)s exchanged with different anion types decrease with an increase in the size of the anions. This decreases results from the larger‐sized anions occupying a substantial portion of the pore space, thereby reducing both the specific surface area and pore volume.

**FIGURE 6:**
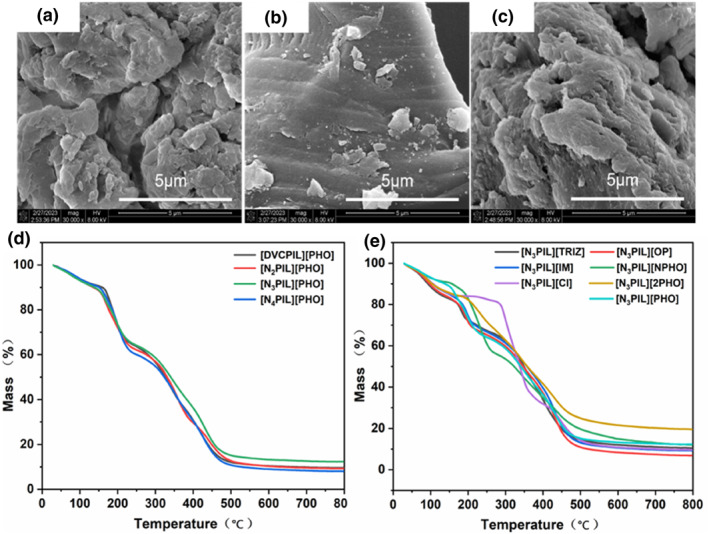
SEM images of (a) [N_3_PIL][PHO], (b) [N_3_PIL][NPHO], (c) [N_3_PIL][Cl]; thermal gravimetric analysis of basic poly(ionic liquid)s [NxPIL][PHO] (d), [N_3_PIL][Y] (e).

Finally, the synthesized poly(ionic liquid)s were subjected to surface morphology and thermal stability analysis, as depicted in Figure 6. The surface of the poly(ionic liquid) appears rough and relatively loose. Such a structure is advantageous for allowing reactants to make sufficient contact with active sites within the poly(ionic liquid), thereby exerting its catalytic function. However, this structure is also prone to adsorbing moisture from the air. As shown in Figure 6, the produced poly(ionic liquid)s exhibit significant weight loss around 100°C and 200°C, attributable to the adsorption of H_2_O and the evaporation of crystalline water. Furthermore, poly(ionic liquid)s with varying numbers of nitrogen‐containing alkaline groups exhibit similar weight loss phenomena at around 300°C, indicating their overall favorable stability.

### 3.2 Catalytic performances

The prepared series of alkaline poly(ionic liquid)s were employed in the transesterification between EC and MeOH. The reaction was conducted at 70°C for 3 h with a catalyst loading of 2 wt.% (based on the mass of EC) under the conditions of *n*(MeOH):*n*(EC) = 10. This evaluation aimed to assess the yield and selectivity of DMC, investigating the impact of doping different ratios of nitrogen‐containing alkaline groups and exchanging various anionic functional groups on the catalytic activity of the transesterification, as indicated in Table 1. Under the same conditions, the catalytic activity of [N_3_PIL][PHO] at a synthesis ratio of 1:3 between [VBC][Cl] and [DVC][Cl] (yielding a DMC yield of 66.11% and a selectivity of 73.04%) was notably higher than that of [N_2_PIL][PHO] (yielding a DMC yield of 58.69% and a selectivity of 67.36%) and [N_4_PIL][PHO] (yielding a DMC yield of 59.36% and a selectivity of 67.00%) at synthesis ratios of 1:2 and 1:4, as well as that of [DVCPIL][PHO], which does not contain nitrogen‐containing alkaline groups (DMC yield of 51.60% and selectivity of 60.78%). This observation, combined with CO_2_‐TPD, surface area and porosity analyses, elemental analysis, and XPS analysis, indicates that the enhanced activity is a result of the doping of nitrogen‐containing alkaline groups, which introduces a stronger alkaline site within the poly(ionic liquid)s. However, excessive doping can block the pore structure of the poly(ionic liquid)s, consequently reducing its specific surface area and hence diminishing the catalytic activity of the transesterification. Furthermore, the catalytic performance of different anionic poly(ionic liquid)s was investigated. In comparison to [N_3_PIL][Cl], the catalytic activity of [N_3_PIL][PHO], [N3PIL][TRIZ], [N_3_PIL][IM], [N3PIL][OP], [N_3_PIL][NPHO], and [N_3_PIL][2PHO] showed a certain degree of enhancement. Among these, [N_3_PIL][PHO] exhibited particularly elevated catalytic activity, establishing itself as a suitable catalytic material.

**TABLE 1:** Comparison of catalytic performance in transesterification of basic poly(ionic liquid)s with different nitrogenous basic group dosing and different anion.

	Catalysts	EC conversion (%)	DMC yield (%)	DMC selectivity (%)
1	[N_2_PIL][PHO]	87.13	58.69	67.36
2	[N_3_PIL][PHO]	90.00	66.11	73.04
3	[N_4_PIL][PHO]	87.82	59.36	67.00
4	[DVCPIL][PHO]	84.89	51.60	60.78
5	[N_3_PIL][TRIZ]	71.93	28.90	40.18
6	[N_3_PIL][IM]	75.47	37.64	49.88
7	[N_3_PIL][OP]	78.91	51.11	64.78
8	[N_3_PIL][NPHO]	68.96	15.58	22.59
9	[N_3_PIL][2PHO]	68.93	13.98	20.28
10	[N_3_FPIL][Cl]	0.00	0.00	0.00

*Note*: Reaction temperature: 70°C, Reaction time: 3 h, Catalyst dosage: 2 wt.%, *n*(MeOH):*n*(EC) = 10.

### 3.3 Effect of reaction conditions on the transesterification of ethylene carbonate with MeOH

**FIGURE 7:**
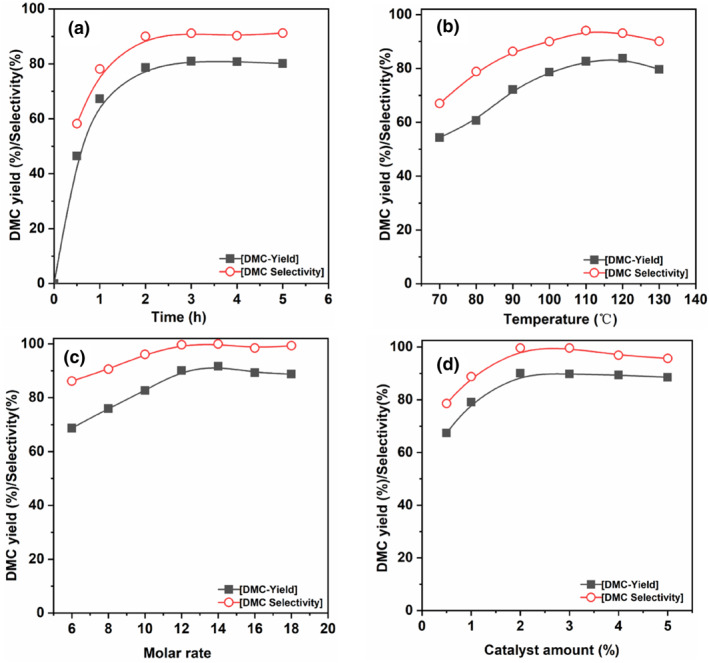
Single‐factor optimization of Dimethyl carbonate (DMC) production with [N_3_PIL][PHO] in transesterification: (a) reaction time (reaction conditions: reaction temperature: 100°C, molar ratio of MeOH to EC: 10:1, catalyst dosage: 2 wt.%); (b) reaction temperature (reaction conditions: molar ratio of MeOH to EC: 10:1, catalyst dosage: 2 wt.%, reaction time: 2 h); (c) molar ratio of MeOH to ethylene carbonate (EC) (reaction conditions: reaction temperature: 110°C, catalyst dosage: 2 wt.%, reaction time: 2 h); (d) catalyst dosage (reaction conditions: reaction temperature: 110°C, molar ratio of MeOH to EC: 12:1, reaction time: 2 h).

The impact of reaction time, temperature, molar ratio of MeOH to EC, and catalyst dosage on the yield of DMC via transesterification catalyzed by [N_3_PIL][PHO] is illustrated in Figure 7. As the reaction time spans from 0.5 to 2 h, the DMC yield escalates from 46.47% to 78.66%, and the selectivity rises from 58.2% to 91.23%. At this point, the reaction approaches equilibrium, with further extension of the reaction time yielding a marginal impact on the DMC yield. Therefore, the optimal reaction time is 2 h. According to Figure 7b, as the reaction temperature ascends from 70°C to 110°C, the DMC yield escalates from 54.31% to 82.66%, and the selectivity rises from 78.85% to 93.07%. This can be attributed to the heightened likelihood of intermolecular collisions with increasing temperature, thus demonstrating an ascending trend in DMC yield within a certain temperature range. However, as the reaction temperature further rose to 130°C, the DMC yield only experienced a marginal increase of 1.1%. This is due to the reaction being slightly exothermic with a small enthalpy change of only 10.52 kJ/mol. Further elevation of the reaction temperature does not correspondingly increase the DMC yield. Consequently, the DMC yield gradually levels off after reaching a peak, indicating an optimal reaction temperature of 110°C.

Furthermore, from Figure 7c, it is apparent that with an increase in the molar ratio of MeOH to EC, the yield of DMC consistently rises. However, at a molar ratio of 12:1, the DMC yield stabilizes at 90.10% with a selectivity of 99.65%. Further increasing the molar ratio of MeOH to EC leads to a reduction in the DMC yield. This phenomenon arises from the excessive quantity of MeOH diluting EC, thereby impacting the interaction between EC and the active sites within the catalytic material. Hence, an increase in the amount of MeOH does not elevate the DMC yield. Consequently, the optimal molar ratio of MeOH to EC is 12:1. Ultimately, the impact of the catalyst [N_3_PIL][PHO] dosage on the performance of EC and MeOH transesterification for DMC synthesis was examined. According to Figure 7d, as the catalyst dosage increases from 0.5 wt.% to 2 wt.%, the DMC yield rises from 61.39% to 90.10%, and the selectivity escalates from 78.57% to 99.65%. As the catalyst dosage continues to increase, the DMC yield stabilizes. This pattern emerges from the fact that augmenting the catalyst dosage enhances the quantity of functional active sites within the system, thereby accelerating the transesterification rate. However, when the quantity of functional active sites reaches saturation, further increasing the catalyst dosage results in uneven dispersion, leading to a less pronounced improvement in its catalytic activity. To conserve costs, the optimal catalyst dosage was determined to be 2 wt.%.

Based on the above results, the appropriate reaction conditions were established: catalyst dosage of 2 wt.%, MeOH to EC molar ratio of 12:1, reaction temperature of 110°C, and reaction time of 2 h. These conditions have been designated as the central points in the orthogonal experimental design for the determination of the optimal conditions.

**FIGURE 8:**
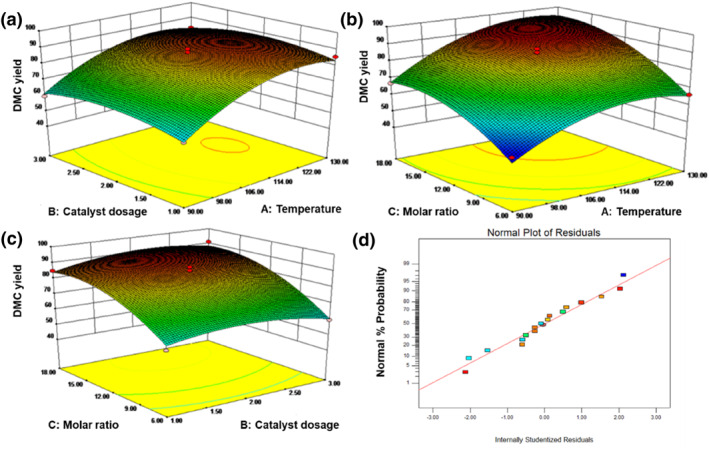
Response surface plot for the yield of Dimethyl carbonate (DMC) from the transesterification of ethylene carbonate (EC) with MeOH by [N_3_PIL][PHO] as a function of: (a) catalyst dosage and reaction temperature, (b) molar ratio of MeOH to EC and reaction temperature, (c) molar ratio of MeOH to EC and catalyst dosage, (d) normal plot of residuals.

Following the results of single‐factor experiments, the operational conditions for the synthesis of DMC through the EC and MeOH catalyzed by [N_3_PIL][PHO] will be further optimized using response surface analysis (Table S6). The results are shown in Figure 8. Among the interactions between the two factors, the 3D surface map with reaction temperature (A) and alcohol ester molar ratio (C) has the steepest degree, indicating that AC interaction has a great influence on DMC yield. The suitable operational conditions for the synthesis of DMC through the EC and MeOH catalyzed by [N_3_PIL][PHO], as verified against the regression model (Equation S1), are as follows: reaction temperature of 119°C, MeOH to EC molar ratio of 13.26:1, catalyst dosage of 2.17 wt.%, with a predicted DMC yield of 92.76%. For practical experimental application, the operational conditions were adjusted to a reaction temperature of 119°C, MeOH to EC molar ratio of 13.00, catalyst dosage of 2.00 wt.%, and reaction time of 2 h. Three experiments were conducted under these adjusted conditions, and the average DMC yield obtained was 91.43% with a selectivity of 99.96%. From these experimental results, it is evident that the obtained values are close to the predicted values, indicating that the established model is capable of effectively predicting the DMC yield.

### 3.4 Mechanism analysis

The swelling behavior of poly(ionic liquid) is attributed to the electrostatic repulsion between the ions along the polymer chains and the imbalance of osmotic pressures inside and outside.[^29^, ^30^, ^31^] This swelling behavior significantly impacts mass transfer during the catalytic process and the enrichment of reactants at the catalytic sites. The swelling properties of poly(ionic liquid)s with varying nitrogen content and anions are shown in Table 2. When poly(ionic liquid)s are doped with varying proportions of nitrogen‐containing alkaline groups, their swelling behavior exhibits an initial increase followed by a decrease, indicating a trend where the swelling properties rise with the increased doping level of nitrogen‐containing alkaline groups, with [N_3_PIL][PHO] showing a higher swelling index (2.60) compared to [N_2_PIL][PHO] and [N_4_PIL][PHO] (2.10, 2.14). This suggests that the doping of nitrogen‐containing alkaline groups significantly affects the affinity of poly(ionic liquid)s for MeOH, consequently influencing their swelling behavior. Moreover, when poly(ionic liquid)s exchange different types of anions, it similarly affects their swelling properties. Considering the catalytic activity results of the transesterification in conjunction with poly(ionic liquid), it is evident that when the poly(ionic liquid) exhibits significant swelling, there is a gradual increase in the activity of the transesterification. Specifically, [N_3_PIL][Cl], lacking alkaline sites, demonstrates lower catalytic efficiency despite its maximal swelling index (6.00). Thus, it is inferred that regulating the cationic structure of poly(ionic liquid) (via doping with nitrogen‐containing alkaline groups) as well as the type of anions can enhance its swelling behavior, thereby facilitating the ingress of reactants into the catalytic material, promoting the interaction between active sites and reactants, and consequently elevating catalytic efficiency.

**TABLE 2:** Swelling degree of poly(ionic liquid)s.

	Catalysts	$V_{\text{dry}}$ (ml)	$V_{\text{wet}}$ (ml)	$Q_{v}$	Yield (%)
1	[N_2_PIL][PHO]	0.50	1.55	2.10	58.69
2	[N_4_PIL][PHO]	0.50	1.57	2.14	59.36
3	[N_3_PIL][PHO]	0.50	1.80	2.60	66.11
4	[N_3_PIL][TRIZ]	0.50	1.89	2.78	28.90
5	[N_3_PIL][IM]	0.50	2.10	3.20	37.64
6	[N_3_PIL][OP]	0.50	1.90	2.80	51.11
7	[N_3_PIL][NPHO]	0.50	0.89	0.78	15.58
8	[N_3_PIL][2PHO]	0.50	0.80	0.60	13.98
9	[N_3_PIL][Cl]	0.50	3.50	6.00	0.00

**FIGURE 9:**
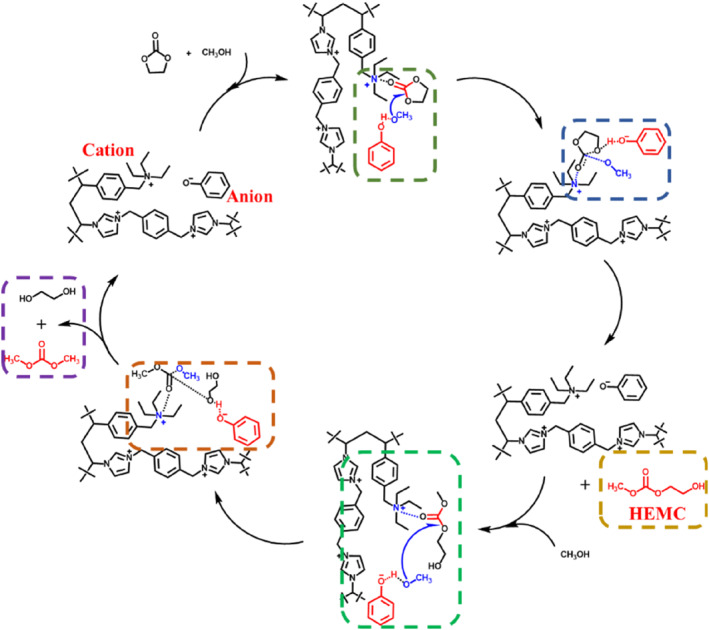
The possible mechanism of the transesterification of EC and MeOH over [N_3_PIL][PHO].

In the analysis of the process for the transesterification synthesis of DMC, Bristow et al.^[32]^ utilized spectroscopy to confirm the generation of the intermediate product hydroxyethyl methyl carbonate (HEMC) during the transesterification between EC and MeOH. Knifton et al.^[33]^ and He et al.^[34]^ similarly substantiated, through their research, that HEMC serves as an intermediate in the alkaline‐catalyzed transesterification synthesis of DMC from EC and MeOH. Based on this, a potential reaction mechanism for the alkaline poly(ionic liquid) [N_3_PIL][PHO] catalyzed EC and MeOH transesterification synthesis of DMC has been proposed (Figure 9). During the transesterification process, the cation and anion of [N_3_PIL][PHO] serve as bridging sites, respectively. The [PHO]^‐^ of the poly(ionic liquid) forms hydrogen bonds with MeOH molecules and gradually abstracts a proton from MeOH, creating the active group CH_3_O^−^. Subsequently, the active group CH_3_O^−^, under the synergistic effect of the cations on the poly(ionic liquid) framework, undergoes nucleophilic attack on the carbonyl carbon atom in EC, causing the cleavage of the C = O bond and forming the intermediate HEMC,^[34]^ as shown in Figure S3. This is followed by further attack by CH_3_O^−^ leading to the ester bond transfer reaction, ultimately producing DMC and ethylene glycol. Meanwhile, the [N_3_PIL]^+^ coordinates with the C = O of EC or HEMC, generating a carbocation via electron transfer and enhancing the electrophilicity of the carbonyl carbon. To verify the mechanism, the ion‐conductivity of poly(ionic liquid)s and yield of the by‐product HEMC with different poly(ionic liquid)s were measured. With [PHO]^‐^ as the anion of the ionic liquid, when the cation part was changed to [N_3_PIL]^+^, the yields of HEMC was reduced to 22.05% (Table S7). Besides, when the cation [N_3_PIL]^+^ was constant and the anion varied, [PHO]^‐^ exhibited the highest activity while the HEMC content was low. These results emphasized the importance of the anion and cation in the transform of HEMC to DMC. The conductivity of poly(ionic liquid) is opposite to the yield of HEMC, and it is related to the binding force of anions and cations. In the transesterification, the yield of DMC increases with the conductivity of poly(ionic liquid) due to the strong dissociation of the anions and cations, which promotes alkalinity. These results suggest that [N_3_PIL]^+^ and [PHO]^‐^ may offer more favorable conditions for enhancing transesterification activity.

### 3.5 Stability and recyclability of the catalyst

**FIGURE 10:**
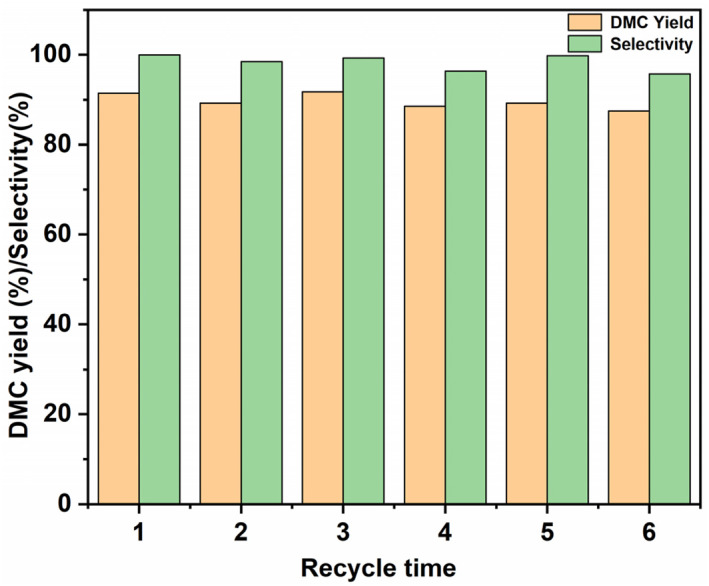
Reusability of [N_3_PIL][PHO] for the transesterification of ethylene carbonate (EC) and MeOH.

To further investigate the structural stability of [N_3_PIL][PHO], an exploration of the material's repeated use stability was conducted under the optimal conditions predicted by the response surface methodology: reaction temperature of 119°C, MeOH to EC molar ratio of 13:1, reaction time of 2 h, and catalyst loading of 2 wt.%. The specific steps are as follows: the raw materials, catalyst, and rotor are added to a pressure‐resistant bottle and the reaction is carried out under the aforementioned optimal conditions. After the reaction is completed, the pressure‐resistant bottle is promptly cooled, and the resulting mixture is sampled and analyzed. After the remaining sample is centrifuged, [N_3_PIL][PHO] is washed with MeOH for three times, and the recovered [N_3_PIL][PHO] is dried using a vacuum freeze dryer. The same proportion of reactants was added back to the pressure‐resistant bottle and the reaction was carried out under the same conditions for 2 h. The above steps were repeated 5 times. The results are shown in Figure 10. After being reused for 5 times, [N_3_PIL][PHO] maintained a DMC yield of 87.55% and a selectivity of 95.71%, indicating that [N_3_PIL][PHO] has excellent catalytic stability, which can be attributed to its suitable cross‐linking skeleton and strong basic sites. Combined with its high catalytic activity and DMC selectivity, it confirms that [N_3_PIL][PHO] is an outstanding transesterification catalyst material.

## 4 CONCLUSION

[N_X_PIL][Cl] with varying nitrogen‐containing alkaline functional groups were prepared by polymerization using nitrogen‐containing alkaline monomers [VBC][Cl] and [DVC][Cl]. [N_X_PIL][PHO] was obtained through anion exchange of [N_X_PIL][Cl] with phenoxide ions. After screening for the most effective catalyst, [N_3_PIL][Cl], the influence of anion type on the transesterification was studied by anion exchange using a variety of anions. The findings indicated that the catalytic activity of the poly(ionic liquid) increased by approximately 15% upon moderate doping of nitrogen‐containing alkaline functional groups. Furthermore, it was confirmed that [N_3_PIL][Cl], which lacks alkaline functional sites, had no catalytic activity, highlighting the crucial role of alkaline functional sites and alkalinity levels of the poly(ionic liquid) in catalyzing the transesterification. The optimal catalytic conditions were obtained through a response surface methodology experiment using [N_3_PIL][PHO] as the catalyst, with a reaction time of 2 h, reaction temperature of 119°C, catalyst dosage of 2 wt.%, and EC to MeOH molar ratio of 1:13. Under these conditions, the predicted yield of DMC was 92.76%. Three independent experiments were conducted under these optimized conditions, and the average DMC yield was found to be 91.43%, which closely matched the predicted value of the response surface model. Through this study, it was concluded that the alkalinity and alkali content of poly(ionic liquid)s can be regulated by adjusting the cation and anion types on the polymer framework, thereby enhancing the reaction performance of transesterification synthesis of DMC.

## CONFLICT OF INTEREST STATEMENT

The authors declare no conflicts of interest.

## ETHICS STATEMENT

The manuscript strictly adheres to ethical guidelines and does not include any experiments involving human subjects or animals. Moreover, all authors have been personally and actively involved in substantive work leading to the manuscript, and will hold themselves jointly and individually responsible for its content.

## Supporting information

Supplementary Material

## Data Availability

The data that support the findings of this study are available from the corresponding author upon reasonable request.
