# Supplementary material for: Functional poly(ionic liquid) with unique zwitterionic structure as efficient catalyst for the conversion of ethylene carbonate to dimethyl carbonate
Source: Smart Mol. 2025 Jan 2;3(4):e20240046. doi: 10.1002/smo.20240046 (PMC12755226; doi:10.1002/smo.20240046)
Supplement: Supplementary file 1 — Supplementary Material [file SMO2-3-e20240046-s001.docx]

Supplementary Material

*for*

Functional poly(ionic liquid) with unique zwitterionic structure as efficient catalyst for the conversion of ethylene carbonate to dimethyl carbonate

Zhaoyang Qi ^1, 2^, Fuying Zhang ^1, 2^, Huiyun Su ^1, 2^, Changshen Ye ^1, 2^, Qinglian Wang ^1, 2, *^, Ting Qiu ^1, 2, 3, *^, Jie Chen ^1, 2^

^1^ College of Chemical Engineering, Fuzhou University, Fuzhou 350108, Fujian, P.R. China;

^2^ Qingyuan Innovation Laboratory, Quanzhou 362801, P.R. China;

^3^ Fuzhou University International Joint Laboratory of Thermochemical Conversion of Biomass.

** Corresponding authors:*

Qinglian Wang, E-mail address: wqlian@fzu.edu.cn.

Ting Qiu, E-mail address: [tingqiu@fzu.edu.cn](mailto:tingqiu@fzu.edu.cn).

**Materials**

The chemicals including methanol (MeOH, CH_3_OH), ethylene carbonate (EC, C_3_H_4_O_3_), divinylbenzene (DVB, C_10_H_10_), 1-Vinylimidazole (C_5_H_6_N_2_), 4-vinyl benzyl chloride (C_9_H_9_Cl), *p*-xylylene dichloride (C_8_H_8_C_l2_), 1,3,5-tris(bromomethyl)benzene (C_9_H_9_Br_3_), triethylamine (C_6_H_15_N), 2,2'-azobis(2-methylpropionitrile) (C_8_H_12_N_4_), sodium hydroxide (NaOH), ethyl alcohol (C_2_H_5_OH), phenol (C_6_H_5_OH), 1,2,4-triazole (C_2_H_3_N_3_), imidazole (C_3_H_4_N_2_), 2-hydroxypyridine (C_5_H_5_NO), nitrophenol (NO_2_C_6_H_4_OH), hydroquinone (C_6_H_6_O_2_), and acetonitrile (C_2_H_3_N) were purchased from Shanghai Aladdin Industrial Corp (China), and used as received without further purification.

**Material characterization**

All samples were activated under vacuum at 100 °C to remove the guest solvents or water. The prepared materials were characterized by nuclear magnetic resonance spectroscopy (^1^H NMR), Fourier transform infrared spectra (FT-IR), elemental analysis (EA), scanning electron microscopy (SEM), X-ray diffraction (XRD), X-ray photoelectron spectroscopy (XPS), thermal gravimetric analysis (TGA) and N_2_ adsorption-desorption isothermals. In the process of ^1^H NMR spectroscopy, the polymeric monomer was dissolved in heavy water or deuterated methanol as a solvent to form a clarified solution, and then analyzed by NMR hydrogen spectrometry. FT-IR measurement was performed on Nicolet 510P FT-IR absorption spectrometer in the range of 400-4000 cm^-1^. Elemental compositions of the catalysts were determined by EA using Vario EL Cube Elementar. The precise method of measurement process of O element involves infusing helium into a high-temperature cracking column housing graphite. This prompts the poly(ionic liquid)s to undergo high-temperature cracking within an inert gas environment, leading to the complete conversion of the O element of poly(ionic liquid)s into CO. Concurrently, other resultant cracked products are either absorbed or isolated while being separated by the helium carrier gas. Ultimately, the CO gas content is gauged using a thermal conductivity detector (TCD). Subsequent processing and computation yield the O content. SEM was performed on Hitachi S-4800 to evaluate the morphology and surface structure of materials. XRD patterns were acquired on Bruker D8 equipped with CuKa radiation (40 kV, 40 mA). The XPS tests were performed on the ESCALAB 250Xi Photoelectron Spectrometer (Thermo Fisher Scientific) equipped with a double-anode target Al-Mg and charge neutralizer. It is used to test the types and valence states of surface elements in catalytic materials. By TGA conducted on Netzsch STA449C, the thermal stability of prepared materials was evaluated. N_2_ sorption isotherms and pore size distributions were collected with Micromeritics automatic analyzer ASAP2020. The pore size distribution was obtained from the adsorption branches of the isotherms using the nonlocal density functional theory (NLDFT) method.

FIGURE S1 The ^1^H NMR spectrum of polymeric monomer [VBC][Cl].


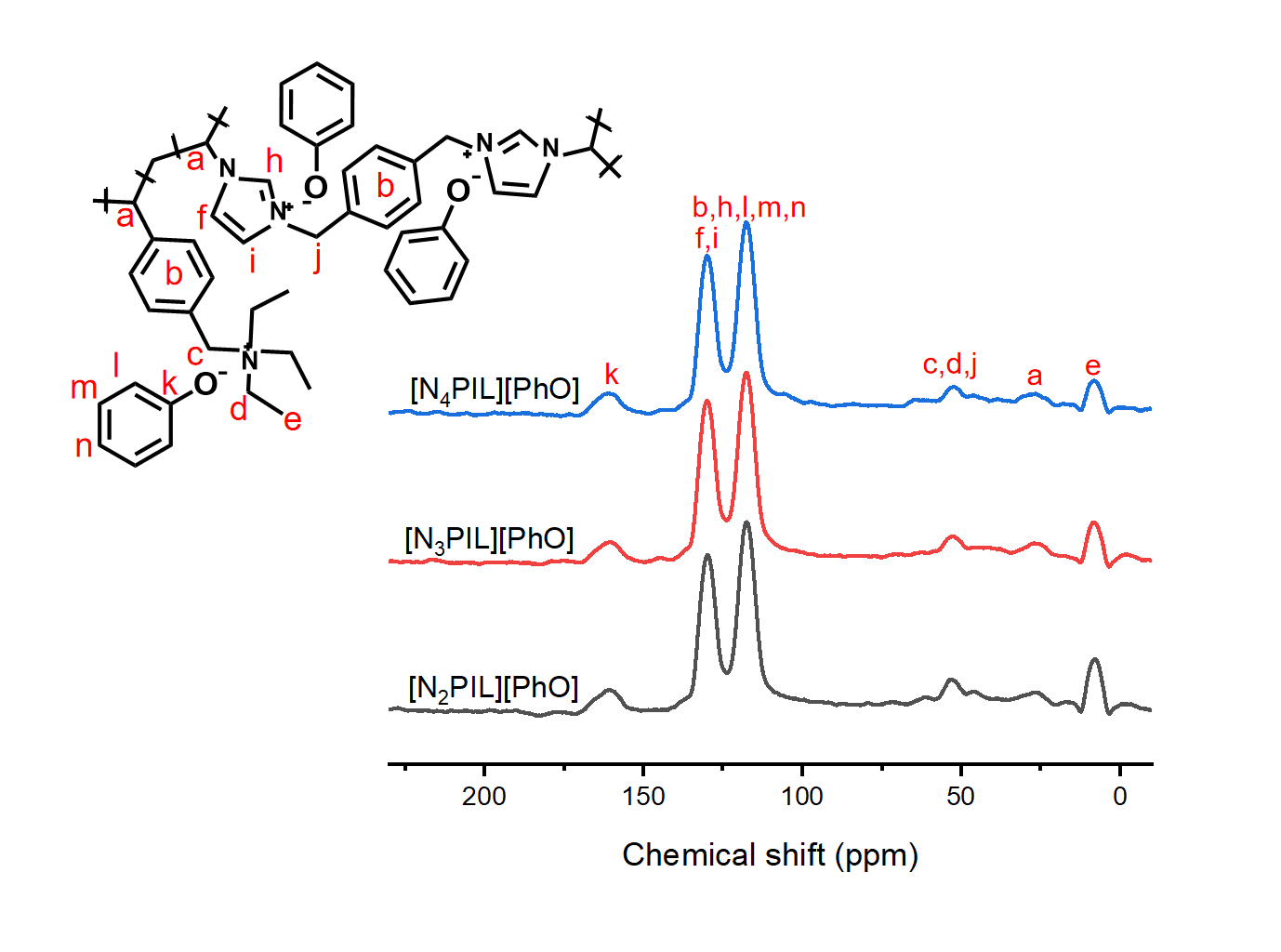


FIGURE S2 ^13^C NMR spectra of [N_2_PIL][PHO], [N_3_PIL][PHO], and [N_4_PIL][PHO].


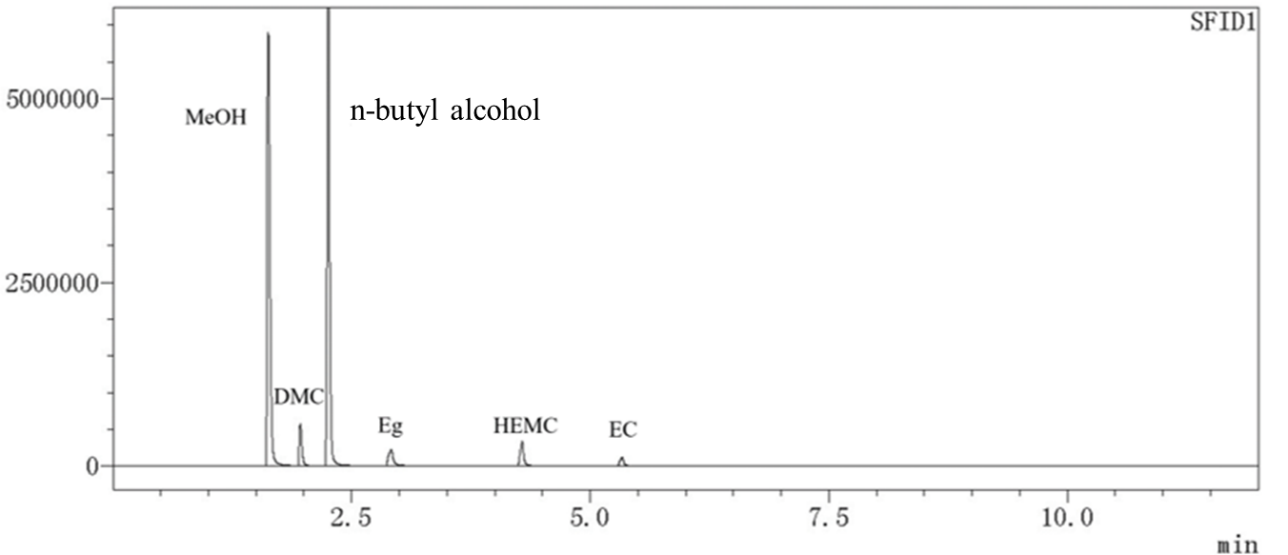


FIGURE S3 The Gas chromatogram of the peak position of each substance during the reaction.

TABLE S1 XPS N 1s spectra information of basic poly(ionic liquid)s [N_2_PIL][PHO], [N_3_PIL][PHO], [N_4_PIL][PHO]

|  | | [N_2_PIL][PHO] | [N_3_PIL][PHO] | [N_4_PIL][PHO] |
| --- | --- | --- | --- | --- |
| C=N-C | Peak (eV) | 398.7 | 398.7 | 398.7 |
|  | Area | 2749.16 | 3843.76 | 2110.04 |
|  | Ratio (*A*_C=N-C_/*A*_sum_) | 0.22 | 0.24 | 0.18 |
| -N^+^- | Peak (eV) | 399.9 | 399.8 | 399.9 |
|  | Area | 2220.67 | 3373.24 | 2212.11 |
|  | Ratio (*A*_-N_^+^_-_/*A*_sum_) | 0.17 | 0.21 | 0.19 |
| C-N^+^ | Peak (eV) | 401.6 | 401.6 | 401.5 |
|  | Area | 7767.87 | 8505.21 | 6891.36 |
|  | (*A*_C-N_^+^/*A*_sum_) | 0.61 | 0.55 | 0.63 |

TABLE S2 CO_2_-TPD peak area product of basic poly(ionic liquid)s [N_2_PIL][PHO], [N_3_PIL][PHO], [N_4_PIL][PHO]

| Samples | Area |
| --- | --- |
| [N_2_PIL][PHO] | 25.01 |
| [N_3_PIL][PHO] | 24.86 |
| [N_4_PIL][PHO] | 22.39 |

TABLE S3 CO_2_-TPD peak area product of basic poly(ionic liquid)s [N_3_PIL][Y]

| Samples | Area | Samples | Area |
| --- | --- | --- | --- |
| [N_3_PIL][PHO] | 24.86 | [N_3_PIL][TRIZ] | 7.96 |
| [N_3_PIL][2PHO] | 28.38 | [N_3_PIL][OP] | 10.69 |
| [N_3_PIL][NPHO] | 15.71 | [N_3_PIL][IM] | 8.55 |

TABLE S4 Surface area, pore volume and pore size of basic poly(ionic liquid)s [N_X_PIL] [PHO]

| Samples | *S*_BET_ (m^2^/g) | Pore volume (cm^2^/g) | Pore size (Å) |
| --- | --- | --- | --- |
| [DVCPIL][PHO] | 35 | 0.052 | > 152 |
| [N_4_PIL][PHO] | 10 | 0.045 | > 409 |
| [N_3_PIL][PHO] | 10 | 0.039 | > 363 |
| [N_2_PIL][PHO] | 5 | 0.034 | > 326 |

TABLE S5 Surface area, pore volume and pore size of poly(ionic liquid)s [N_3_PIL][Y]

| Samples | *S*_BET_ (m^2^/g) | Pore volume (cm^2^/g) | Pore size (Å) |
| --- | --- | --- | --- |
| [N_3_PIL][PHO] | 10 | 0.039 | > 363 |
| [N_3_PIL][TRIZ] | 12 | 0.044 | > 487 |
| [N_3_PIL][OP] | 11 | 0.045 | > 378 |
| [N_3_PIL][IM] | 7 | 0.041 | > 581 |
| [N_3_PIL][2PHO] | 1 | 0.045 | > 330 |
| [N_3_PIL][Cl] | 15 | 0.055 | > 645 |
| [N_3_PIL][NPHO] | 1 | 0.001 | > 370 |

TABLE S6 Response surface analysis with factors and levels of transesterification

| Factors | Symbol | Level and range | | |
| --- | --- | --- | --- | --- |
|  |  | -1 | 0 | 1 |
| Reaction temperature /℃ | *A* | 90 | 110 | 130 |
| Catalyst dosage | *B* | 1 | 2 | 3 |
| Molar ratio | *C* | 6 | 12 | 18 |

TABLE S7 The results for the poly(ionic liquid) catalyzed transesteriﬁcation reaction of EC with MeOH

|  | Catalysts | EC conversion  (%) | DMC yield  (%) | HMEC yield  (%) | σ_DC_×10^-4^  /(S·cm^-1^) |
| --- | --- | --- | --- | --- | --- |
| 1 | [N_2_PIL][PHO] | 87.13 | 58.69 | 27.12 | 0.35 |
| 2 | [N_3_PIL][PHO] | 90.00 | 66.11 | 22.05 | 0.43 |
| 3 | [N_4_PIL][PHO] | 87.82 | 59.36 | 26.52 | 0.36 |
| 4 | [N_3_PIL][TRIZ] | 71.93 | 28.90 | 39.18 | 0.18 |
| 5 | [N_3_PIL][IM] | 75.47 | 37.64 | 36.52 | 0.20 |
| 6 | [N_3_PIL][OP] | 78.91 | 51.11 | 25.32 | 0.36 |

Reaction temperature: 70 ℃, Reaction time: 3 h, Catalyst dosage: 2 wt.%, *n*(MeOH):*n*(EC)=10

$\text{Y}_{\text{DMC}}\text{ = 87.33 + 11.3}\text{A}\text{ + 0.44}\text{B}\text{ + 12.37}\text{C}\text{ - 0.57}\text{AB}\text{ + 2.22}\text{AC}\text{ + 1.63}\text{BC }\text{- 10.30}\text{A}^{\text{2}}\text{- 5.19}\text{B}^{\text{2}} \text{- 8.33}\text{C}^{\text{2}}$ （Equation S1）
